# The Association Between Hematological Profiles and Whole-Blood Transcriptome Genes Identified Using Quantitative Analysis with Average Daily Gain and Feed Efficiency in Forage-Fed Beef Heifers †

**DOI:** 10.3390/ijms26104633

**Published:** 2025-05-13

**Authors:** Amanda K. Lindholm-Perry, Heather L. Bradford, Andrew P. Foote, Harvey C. Freetly, Carol G. Chitko-McKown, Larry A. Kuehn, John W. Keele, Bryan W. Neville, William T. Oliver, Brittney N. Keel

**Affiliations:** 1 USDA-ARS, U.S. Meat Animal Research Center, Clay Center, NE 68933, USA; amanda.lindholm@usda.gov (A.K.L.-P.); heather.bradford@usda.gov (H.L.B.); harvey.freetly@usda.gov (H.C.F.); carol.chitkomckown@usda.gov (C.G.C.-M.); larry.kuehn@usda.gov (L.A.K.); john.keele@usda.gov (J.W.K.); bryan.neville@usda.gov (B.W.N.); william.oliver@usda.gov (W.T.O.); 2 Department of Animal and Food Sciences, Oklahoma State University, Stillwater, OK 74078, USA; andrew.foote@okstate.edu

**Keywords:** feed efficiency, whole-blood transcriptome, gene expression, cattle

## Abstract

Feed is the single greatest cost for cattle producers. Improvements to feed efficiency, or how animals convert feed to body weight gain, will ultimately improve producer profits. The objective of this study was to determine whether the expression of genes in the transcriptome of whole blood from heifers (*n* = 61) on a forage ration was related to the quantitative phenotypes for average daily gain (ADG), average daily feed intake (ADFI), and gain-to-feed (G:F). Total RNA was isolated from whole blood collected mid-study on day 42 on feed and was used for hematologic analysis and RNA-sequencing. Lymphocyte (LYM) count was negatively associated with ADG, and mean corpuscular hemoglobin concentration (MCHC) was positively associated (*p* < 0.05). Red blood cell (RBC) count was negatively associated with ADFI. While MCHC was positively associated with G:F, white blood cell (WBC), LYM, and basophil (BAS) counts were negatively associated with G:F (*p* ≤ 0.05). The model used to identify differentially expressed genes (DEG) for ADFI, ADG, and G:F included sire, breed, pen, age, and proportions of blood cell types. No genes were differentially expressed for ADFI. Three genes were identified as differentially expressed for ADG, and 17 were identified for G:F. Three of the differentially expressed genes for G:F and ADG (*PLOD1*, *FAH*, and *COL1A2*) had been previously associated with feed efficiency in livestock and may be useful for further validation in other populations of cattle. The negative associations between WBC and LYM and ADG and G:F may be expected since the production of WBC is an energetic process that may reduce body weight gain and efficiency. The associations between RBC hematological parameters suggest that there may be benefit for animals with higher levels of hemoglobin per RBC by improving oxygen-carrying capacity.

## 1. Introduction

For producers, feed is the single largest expense for heifers from birth to calving and has been calculated at 73% of total production costs [1]. Improvements in feed efficiency will reduce the amount of feed consumed and have the potential to drastically reduce feed input costs for producers. Feed efficiency in beef heifers is strongly correlated with the feed efficiency of the same animals as mature cows [2]. In this study, we measured individual animal feed intake and body weight gain (the component traits of feed efficiency) in heifers consuming a forage diet to determine whether differences between animals could be associated with genes expressed in whole blood using Ribonucleic acid sequencing (RNA-seq). These genes may help us to identify mechanisms and pathways that contribute to differences in feed efficiency, which may serve as new options for genetic selection or targets for the improvement of feed efficiency. Whole blood was selected because blood is a relatively non-invasive sample to obtain and is easily collected from cattle. Moreover, gene expression from whole blood has been associated with feed efficiency in prior studies [3,4,5,6].

Most studies that employ RNA-seq methods select animals with the most extreme phenotypic measurements. While costs to perform RNA-seq are decreasing, it is still relatively expensive to generate mRNA libraries for each animal/sample, and there are additional costs to obtain enough sequence coverage to identify differences in gene expression. There are also issues with the collection of tissues that are likely to be involved in feed efficiency (i.e., muscle, rumen, liver, etc.), which often require either semi-invasive biopsies or harvesting the animal for collection. The result is often the comparison of 4–16 animals per phenotypic group, and the data analysis is carried out using a categorical value (i.e., high or low feed efficiency). This type of experimental design likely fails to capture all the variation in gene expression that contributes to these phenotypes.

We hypothesized that gene expression in whole blood is associated with average daily gain (ADG), average daily feed intake (ADFI), and gain-to-feed (G:F). For this study, we chose to perform RNA-seq on all heifers fed a forage diet during a feed efficiency study, and we analyzed the data using the quantitative phenotypes for ADG, ADFI, and G:F. The inclusion of all animals in the study reduces potential bias that can occur from any one animal used in smaller categorical trait analyses. Additionally, the quantitative phenotype should produce more robust differentially expressed gene (DEG) results. However, whole blood includes a complex mix of cell types that can confound transcriptome results. To circumvent this, we included the reference hematology profiles in the analysis to account for differences in cell types among animals that may influence gene expression results.

## 2. Results

### 2.1. Feed Efficiency Phenotypes and Hematology Parameters

Feed efficiency phenotypes for the 61 heifers are presented in Table 1. Average weights increased from 283 kg (SD, ± 3.491 kg) on d 0 to 347.4 (SD, ± 4.377 kg) on d 84. Averages for ADG, ADFI, and G:F were 0.8 kg/d, 7.5 kg/d, and 0.1 kg gain/kg intake, respectively. Whole-blood samples taken on d 42 of the study were analyzed on a veterinary hematology instrument. Average values and standard deviations, along with minimum and maximums, for the fifteen measured blood parameters are presented in Table 2.

### 2.2. Phenotype Associations with Hematology Parameters

Baseline *R*^2^ variation from models including only breed, age, pen, and sire was 0.1941 for ADG, 0.2241 for ADFI, and 0.1113 for G:F. White blood cell (WBC), lymphocyte (LYM), basophil (BAS), and red blood cell (RBC) counts were negatively associated with ADG and accounted for 0.96% to 5.85% more variation, and mean corpuscular hemoglobin (MCH) and mean corpuscular hemoglobin concentration (MCHC) were positively associated with ADG, explaining an additional 2.1% and 11.66% of the variation in ADG, respectively (*p* < 0.1; Table 3). RBC and hematocrit (HCT) were negatively associated with ADFI and accounted for an additional 6.44% and 5.19% of the variation in ADFI, respectively. MCHC was positively associated with ADFI, explaining an additional 8.21% of the variation. WBC, LYM, and BAS were negatively associated with G:F, explaining an additional 4.57% to 13.98% of the variation. MCH and MCHC were negatively associated with G:F, explaining an additional 2.79% and 6.42% of the variation, respectively.

### 2.3. Differentially Expressed Genes for ADG and ADFI

**Figure 1:**
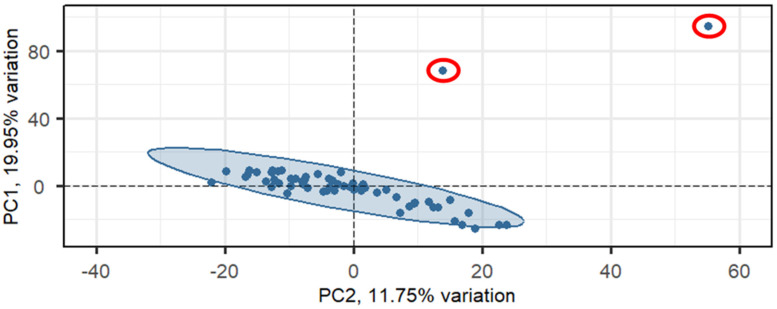
Principal component analysis (PCA) plot of normalized gene expression values. Outlier samples (circled in red), located outside of the 98.5% confidence ellipse shown, were removed from differential expression analysis.

Gene expression levels in blood, collected from the 61 heifers, were quantified using RNA-seq. An average of 40.2 million reads were generated per library (Table S1). After trimming, sequence reads were mapped to the Bos taurus ARS-UCD1.2 genome assembly with an average 90.72% read mapping rate. On average, 77.41% of the reads mapped to gene regions, with 0.08% of reads mapping to hemoglobin genes. After filtering lowly expressed genes, a total of 15,315 of the 33,845 genes in the cattle genome remained for downstream analysis. PCA identified two outlier samples that were removed prior to differential expression analysis (Figure 1).

Given the observed differences in cell counts relative to the feed efficiency phenotypes, adjustments for the concentration of blood cell types and confounding factors (pen, age, breed, and sire) were made to both gene expression and phenotype prior to differential expression analysis. Table 4 shows the differentially expressed genes (DEG) associated with ADG and G:F (False discover rate (FDR)-adjusted-*p* < 0.1). No DEGs were associated with ADFI.

Three DEGs were found to be associated with ADG, including *FAH*, *LOC104972586*, and *COL1A2* (Table 4). Expression of *COL1A2* was positively associated with ADG, while *FAH* and *LOC104972586* exhibited negative associations with ADG. These genes did not cluster by either function or pathways.

A total of 17 DEG were associated with G:F (Table 4). Expression of *COL1A2*, *B9D1*, *CCDC151*, *NEK2*, *CIAPIN1*, *U2AF1*, *XKR5*, *ATP6V0E2*, *UQCRFS1*, *LOC112443184*, and *BCL2A1* was positively associated with G:F, and expression of *SMARCA2*, *RGS10*, *NRP2*, *PLOD1*, *FAH*, and *LOC101904536* was negatively associated with G:F. These genes did not cluster into known biological processes or cellular component GO terms; however, the molecular functions metal ion binding and 2 iron, 2 sulfur cluster binding were over-represented by the genes in this list. The term metabolic pathways was the only pathway identified using the list of differentially expressed genes.

## 3. Discussion

We have performed several transcriptome studies in various tissues that are likely to contribute to feed efficiency in beef steers [7,8,9,10,11]. In these studies, we identified several different genes and pathways that appear to play roles in feed efficiency; however, the ability to perform large studies on a tissue sample requiring invasive collection, like rumen or semi-invasive collection like liver, muscle, and fat depots, can restrict the number of animals sampled and studied. Recently, Basu et al. [12] illustrated successful gene expression predictions for tissues based on the transcriptome of blood in humans, suggesting a relationship between various tissues and circulating blood cells. Additionally, studies using whole blood in pigs and cattle have shown gene expression and hematologic parameter differences in animals with variation in feed efficiency phenotypes [3,4,5,6,13,14,15]. Blood is a much less invasive sample to obtain and does not require animals to be euthanized or biopsied; thus, the major aim of this study was to determine whether whole blood could be used to identify genes with expression associated with ADG, ADFI, and G:F.

Compared to many of the aforementioned studies using the whole-blood transcriptome to investigate biomarkers for feed efficiency, the current study is novel in that blood samples for gene expression analysis were collected from all animals in the study, rather than selecting a subset of animals with extreme phenotypes. This allowed us to use continuous phenotypes in the gene expression analysis instead of relying on categorical phenotypes. Accounting for the continuous distribution of quantitative traits should improve statistical power and reduce information loss, because dichotomizing a continuous biological process may homogenize individuals together who lie on a continuum of the trait of interest [16].

A recognized hindrance to whole blood transcriptomics is the presence of large quantities of globin mRNA transcripts in peripheral blood [17], a consequence of abundant α and β globin mRNA transcripts in circulating reticulocytes, which, in humans, may account for more than 95% of the total cellular messenger ribonucleic acid (mRNA) content [18]. This has prompted the development of methods for the systematic reduction of globin mRNA from total RNA samples purified from peripheral blood [19], as well as methods for bioinformatic depletion of hemoglobin reads [20,21]. Bovine peripheral blood samples have been shown to exhibit extremely low proportions of hemoglobin gene transcripts to total expressed genes, typically less than 1% [22,23], suggesting that depletion of globin mRNA transcripts is not a pre-requisite for transcriptome profiling in cattle [23]. In this study, hemoglobin depletion was not conducted during library preparation, and similar hemoglobin proportions of ~0.08% of the reads in each library were observed (Table S1).

Another potential complication for the RNA-seq analysis of blood samples is cellular heterogeneity. Gene expression levels measured from bulk heterogeneous mixtures only represent averaged expression levels, and thus, differential expression analyses are often confounded by differences in cell type proportions [24]. In this work, we used cell counts, measured using CBC, to account for cell type proportion heterogeneity across blood samples. Blood cell composition was directly associated with the measured phenotypes, with WBC, LYM, BAS, and RBC associated with ADG; WBC, LYM, and BAS associated with G:F; and RBC associated with ADFI (Table 3). These findings support the usefulness of considering cell composition when profiling the transcriptome of heterogeneous tissues.

In this study, we show that lower levels of WBC, LYM, and BAS were associated with higher ADG and G:F. Other studies of hematologic associations with feed efficiency have shown results that contradict ours. In a prior study by Consolo et al. [15], the authors identified higher LYM and lower NEU in efficient heifers in a grass-fed feed efficiency study. Foote et al. [25] detected positive associations between LYM and ADG, ADFI, and G:F in heifers. Of these studies, the one presented herein included the fewest animals (*n* = 59), compared to Consolo et al. [15] with 107 animals and Foote et al. [25] with 109 animals. The differences in these studies may be, at least partly, explained by differences in experimental/environmental conditions. For example, the two studies on heifers from USMARC in Nebraska were performed in different years, and the study by Consolo et al. [15] was performed in Canada. Differences in climate, seasonal temperatures, and exposure to pathogens may have contributed to the differences in WBC sub-population concentrations. It is important to note that while none of the animals in this study had WBC counts outside of the normal range, which suggests that infection had a minimal impact on the study, it is still feasible that stress of variation in immune status may play a role and have been a contributing factor in this study.

In the present study, we identified RBC count as negatively associated with both ADFI and a tendency for negative association with ADG (i.e., fewer RBCs with higher ADFI and ADG). Mean corpuscular hemoglobin concentration (MCHC) was positively associated with ADG and ADFI and tended to be associated with G:F. Additionally, a trend was detected with mean corpuscular hemoglobin (MCH) and ADG and G:F (*p* < 0.1). Mean cell hemoglobin is a measurement of the amount of hemoglobin in RBCs, and MCHC is the mass of hemoglobin per RBC. A prior study on heifer calves classified as efficient using residual feed intake (RFI) showed that they had lower mean cell hemoglobin (MCH) concentrations than inefficient animals [15]. Consolo et al. [15] hypothesized that inefficient animals have greater oxygen requirements due to increased heat production [26], higher basal metabolic rates, or that they may be more active [27] than efficient animals. The results by Consolo et al. [15] appear to contradict the results of this study, wherein MCH was positively associated with ADG and G:F.

In humans, an increase in hemoglobin is associated with the need for additional oxygen-carrying capacity. For example, higher hemoglobin levels are associated with changes in altitude when greater oxygen-carrying capacity is needed due to the lower oxygen levels at higher altitude [28]. In several studies, hemoglobin and hematocrit levels in swine, sheep, and cattle have been associated with feed efficiency, ADG, and/or ADFI [6,14,29]. These studies also suggest that these phenotypes may be associated with a need for higher or lower oxygen-carrying capacity. Jegou et al. [14] identified higher RBC count, hemoglobin, and hematocrit in pigs with lower residual feed intake (RFI). Another prior study in swine showed a positive association between increases in RBC count, hemoglobin, and hematocrit with ADG and ADFI over the course of a feeding trial [6]. The results of a study by Quintero-Gutierrez et al. [30] showed that animals fed a higher level of heme iron supplemented in biscuit filling also had greater weight gain and reduced mortality than control pigs. These studies indicate that there may be a body weight production benefit for animals with higher levels of hemoglobin or a higher level of hemoglobin in their RBCs.

In the present study, we identified the gene *fumarylacetoacetate hydrolase* (*FAH*) as negatively associated with both ADG and G:F. FAH is an enzyme that breaks down the amino acids tyrosine and phenylalanine. It functions in the last step of the tyrosine catabolism pathway. Mutations in the *FAH* gene that lead to the abolishment of enzyme activity in humans can result in a condition called tyrosinemia. Tyrosinemia is a result of the build-up of toxic tyrosine intermediary metabolic compounds, which presents as low growth rates. The authors of a prior study on the liver proteome of animals with high and low feed efficiency [31] identified higher concentrations of FAH in low feed efficiency animals. They postulate that FAH, as an anti-oxidation protein, may have higher expression in low feed efficiency animals due to an increase in oxidative stress. Alternatively, these animals may not be able to sequester enough oxygen to meet their metabolic needs. In this study, higher expression of *FAH* was associated with lower BW gain and G:F, which would correspond to cattle with lower feed efficiency and support the findings of [31].

Two genes involved in collagen and its formation were identified as differentially expressed for G:F. These were the *collagen type I alpha 2 chain* (*COL1A2*) and *procollagen*-*lysine,2-oxoglutarate 5-dioxygenase 1* (*PLOD1*). The *COL1A2* gene codes for the alpha chain of type 1 collagen in connective tissue, and the PLOD1 protein is responsible for catalyzing the hydroxylation of lysine in the alpha chain of collagen and is necessary for normal collagen assembly and cross-linking. A study by Banerjee et al. [32] showed that genetic variants in *PLOD1* were associated with lysine degradation in pigs that were considered low feed efficient (inefficient) animals. Lysine deficiency negatively impacts an animal’s immunity and growth [33]. In the current study, *PLOD1* was more highly expressed in animals with lower G:F (less efficient), whereas *COL1A2* was more highly expressed in animals with higher G:F (more efficient animals). Increases in *PLOD1* may allow the conversion of more lysine into collagen chains, resulting in less lysine to support animal growth and health. The *COL1A2* gene was identified in a co-expression network analysis as associated with residual feed intake in Nellore cattle [34]. The same group had previously identified *COL1A2* as upregulated in the liver and muscle tissue of cattle with low RFI [35,36], of which the direction of expression agrees with this study in the whole blood.

Several genes with functions in apoptosis were also identified for G:F. These genes included *CIAPIN1*, *XKR5*, and *BCL2A1*, and all were more highly expressed in animals with higher G:F. To our knowledge, none of these have been identified in other studies examining feed efficiency in livestock. The cytokine-induced gene *CIAPIN1* inhibits apoptosis and might suggest a role in maintaining circulatory cells that may otherwise be slated for cell death. The BCL2A1 protein product has cytoprotective functions and is a direct transcriptional target of the NF-kappa B, which is induced by inflammatory mediators and may ultimately preserve cells exposed to inflammatory signals.

While there are other studies that have evaluated the whole-blood or WBC transcriptome in livestock with variation in feed efficiency phenotypes [14,37,38], none have accounted for variation in cell counts as we have in this study, thereby making comparisons between studies challenging. Changes in gene expression from studies that do not account for differences among animals in cell populations could identify expression levels associated with variation in cell type counts.

The genes identified in this study, for the most part, did not have overlapping functions and did not appear to be from common pathways. Part of our hypothesis was that using phenotype as a quantitative trait on all animals in the study would allow us to identify genes with expression that are either positively or negatively associated with ADG and ADFI. While this was true for the small number of genes identified for ADG and G:F, we did not detect any genes for ADFI. Complex traits, like gain and feed intake, are thought to be controlled by small effects of many genes. A large amount of variation among these genes could make it a challenge to detect specific genes driving these traits over a larger group of animals. We have also shown that genes identified as differentially expressed in one group of animals may be associated in another group with the opposite direction of expression [11,39]. This could be contributing to the challenge of identifying genes that increase or decrease with phenotype across a population of animals. Moreover, it is possible that environmental, management, breed, and/or behavior issues increase the complexity of gene expression signals in the whole blood, which may make validation in other populations of cattle challenging.

## 4. Materials and Methods

### 4.1. Institutional Animal Care and Use

All use and handling of animal subjects were approved by the U.S. Meat Animal Research Center’s Institutional Animal Care and Use Committee (Experiment number: 43.1, approved on 11 May 2017).

### 4.2. Animal Population and Sampling

A total of 80 heifers that were progeny of artificial insemination from industry sires (*n* = 12) from the U.S. Meat Animal Research Center Weight Trait Project, representing Red Angus, Charolais, and Simmental breeds, were used for this project. At weaning (~6 months of age), calves were allowed to consume a receiving diet ad libitum for three weeks and were then transitioned to a forage-based growing diet, consisting of 30.0% chopped alfalfa hay, 69.8% corn silage, and 0.2% salt based on dry matter (DM), which was fed to the heifers throughout the study. Approximately seven weeks after weaning, the calves were moved to a barn with Calan gates (American Calan, Northwood, NH, USA) to measure ad libitum individual feed intakes. The calves were stratified and penned by weight and allowed to acclimate to the gates for three weeks. Those that failed to train were removed from the study. Heifers received Revalor-IH (Merck & Co., Inc., Rahway, NJ, USA) prior to the beginning of the study. The cattle were fed once daily throughout the experiment. Feed was subsampled daily, and a weekly composite sample was collected. Feed refusals (orts) were collected once per week. Total feed intake was derived as the amount of feed delivered minus orts. Average daily feed intake was computed as total feed intake divided by 84 d. Body weights (BW) were collected every 3 weeks throughout the study, with double weights collected at the beginning and the end of the 84-day trial (on d 0 and 1 and on d 83 and 84), and a quadratic equation was used to regress BW on the day of study. Total BW gain was determined by solving the equation for d 84 and subtracting the initial BW. ADG was computed as total BW gain divided by 84 d. ADG was divided by ADFI to determine the gain-to-feed (G:F) phenotype for each of the heifers.

On d 42, a whole-blood sample was collected in a Tempus™ Blood RNA tube (Thermo Fisher Scientific, Detroit, MI, USA) for RNA stabilization and inserted into a tube containing ethylenediaminetetraacetic acid (EDTA) as an anticoagulant for hematologic analysis. Samples were collected from the 61 heifers that remained in the study at d 42. The samples were stored on ice until received at the laboratory within 1 to 3 h of collection, at which time the samples in EDTA were evaluated on a Heska HT5 veterinary hematology analyzer (Heska, Loveland, CO, USA), and the samples in Tempus tubes were stored in a −20 °C freezer until further processing.

### 4.3. Hematology Parameter Measurement

Whole-blood samples in EDTA tubes were placed on gentle rotation on a tube rocker at room temperature until analysis. Samples were tested for white blood cell (WBC) and red blood cell (RBC) hematologic parameters. Hematologic parameters included WBC counts, neutrophil (NEU), lymphocyte (LYM), monocyte (MONO), eosinophil (EOS) and basophil (BAS) counts and percentages, RBC counts, hemoglobin, hematocrit, mean corpuscular volume (MCV), mean corpuscular hemoglobin (MCH), mean corpuscular hemoglobin concentration (MCHC), platelet count, and mean platelet volume (MPV).

### 4.4. Phenotypic Association with Hematology Parameters

Hematologic parameters were tested for effects on ADG, ADFI, and G:F using individual linear mixed models that included breed and pen as fixed effects, sire as a random effect, and age and the hematologic parameter as covariates. Model *R*^2^ was compared to baseline models for ADG, ADFI, and G:F that included only breed, age, pen, and sire to determine the amount of additional variation explained by the addition of the hematology covariates. Mixed model analysis was conducted using the lmer function in R [40]. Prior to mixed model analysis, singular value decomposition was used to ensure no linear dependencies between terms in the baseline model.

### 4.5. RNA Isolation and RNA-Seq Library Preparation

Total RNA was isolated from the 61 whole-blood samples in Tempus tubes using the Tempus™ Spin RNA Isolation Kit (ThermoFisher Scientific, Waltham, MA, USA) following the manufacturer’s protocol. A subset of 12 RNA samples was evaluated on an Agilent Bioanalyzer and had an average RIN of 9.6 (range of 9.1 to 10). The concentration of RNA was determined using a Nanodrop ONE spectrophotometer (ThermoFisher Scientific). All samples yielded A260/280 ratios of ≥1.8. Libraries for RNA-sequencing were prepared with the TruSeq Stranded mRNA library prep kit according to the manufacturer’s instructions using 500 ng of total RNA. The libraries were sequenced on an Illumina NextSeq (Illumina, San Diego, CA, USA) instrument with a 150-cycle sequencing kit (2 × 75 bp reads) in pools of 24 to 26 libraries.

### 4.6. RNA-Seq Read Processing

The quality of the raw paired-end sequence reads in individual fastq files was assessed using FastQC (Version 0.11.5; www.bioinformatics.babraham.ac.uk/projects/fastqc (accessed on 4 February 2019)), and reads were trimmed to remove adapter sequences and low-quality bases using fastp software (Version 0.23.2) [41] with the default parameters and the -trim_poly_x option. Remaining reads were mapped to the ARS-UCD1.2 genome assembly (NCBI Refseq Accession GCF_002263795.1) using Hisat2 (Version 2.1.0) [42]. The NCBI annotation for ARS-UCD1.2 (Release 106) was used to guide the alignment. StringTie (Version 2.2.1) [43] was used to determine read counts for each of the annotated genes in the ARS-UCD1.2 genome assembly. The raw sequencing data can be accessed in the NCBI Sequence Read Archive (SRA) database with accession number PRJNA1153552.

### 4.7. Differential Gene Expression Analysis

Prior to differential expression analysis, genes that had an average read count <10 across all samples and a zero read count in >80% of the samples were removed. Gene expression values were normalized using DESeq2 (Version 1.36.0) [44], and principal component analysis (PCA) was performed on normalized gene expression values, removing the lower 10% of genes based on variance. PCA analysis was conducted using the PCAtools package in R (Version 2.6.0) [45]. A 98.5% confidence ellipse was constructed in the plane determined using the first 2 principal components. Two outlier samples, located outside the confidence ellipse, were removed, leaving a total of 59 samples for differential expression analysis.

Normalized gene expression values and traits (ADG, ADFI, or G:F) were adjusted for confounders using linear mixed models that included breed and pen as fixed effects, sire as a random effect, and age and proportions of blood cell types (NEU, LYM, MONO, EOS, BAS, and RBCs) as covariates. Mixed models were run using the lmer function in R. Residuals from the mixed models were extracted and used as the adjusted values for differential expression analysis. For each of the three traits, the Pearson correlation between adjusted gene expression and the adjusted trait was calculated on a gene-by-gene basis. *p*-values were adjusted for false discovery rate (FDR) using the Benjamini–Hochberg method. Genes with FDR-adjusted *p*-values < 0.1 were considered statistically significant.

### 4.8. Gene Ontology and Pathway Analysis

iPathwayGuide software (Version 17.1; https://advaitabio.com) (accessed on 25 June 2024) was used to identify significantly enriched gene ontology (GO) terms and biological pathways in the DEGs.

## 5. Conclusions

Whole blood might not reflect the expression of genes that are critical for ADG and G:F among tissues that would be expected to influence these phenotypes, like liver, muscle, adipose, etc., as we hypothesized. In other words, whole blood may be a poor proxy for the genes influencing feed efficiency in various tissues. While other studies have shown an association between gene expression with feed efficiency in livestock, none that we know of have performed a quantitative analysis including effects, like sire, pen, age, and breed, as well as the composition of cell types. The analysis presented herein could be over-parameterized and/or may not be suitable for traits with large variation among individuals or with genes that have small effects.

In summary, in a group of heifers in a forage-fed efficiency study, we identified negative associations between WBC hematologic parameters and ADG and G:F. The production of more WBCs is likely to have an energetic cost, thus decreasing the pool of nutrients and energy available for production and weight gain. Our results also indicated that increasing numbers of RBCs are associated with decreased ADFI and may show that when oxygen-carrying capacity is low, more RBCs are needed to provide oxygen for metabolic performance. Additionally, if RBCs have higher levels of hemoglobin available for oxygen transport, fewer may be needed for the animal to operate more efficiently. Finally, we detected associations between ADG and G:F phenotypes and a few genes that were differentially expressed in the whole blood of these cattle. We hypothesized that the association between gene expression and complex phenotypes should be improved with larger numbers of animals and a model that accounts for variation in whole-blood cell types. While we did detect a few genes that were associated with ADG and G:F, we did not detect any genes differentially expressed for ADFI. A few of the genes identified for ADG and/or G:F had been previously identified in the muscle and liver of animals with variation in feed efficiency. These included *FAH*, *PLOD1*, and *COL1A2*, and the validation of these genes with gain and G:F in additional populations of animals may be warranted.

## Figures and Tables

**Table 1:** Feed efficiency phenotypic information from all beef heifers (*n* = 61).

Phenotype ^1^	Average (SD)	Minimum	Maximum
Weight (d 0)	283.0 (27.3)	228.6	356.5
Weight (d 21)	306.5 (26.7)	250.4	374.7
Weight (d 42)	315.2 (29.2)	259.5	404.6
Weight (d 63)	333.5 (32.1)	270.3	424.6
Weight (d 84)	347.4 (34.2)	286.7	436.4
ADG	0.8 (0.2)	0.3	1.4
ADFI	7.5 (1.2)	5.1	11.0
G:F	0.11 (0.03)	0.05	0.16

^1^ Weights are expressed in kg, average daily gain (ADG) and average daily feed intake (ADFI) are expressed in kg/d, and gain-to-feed (G:F) is expressed in kg gain/kg feed.

**Table 2:** Hematology parameters for all beef heifers (*n* = 61) measured on d 42 of the study.

Parameter ^1^	Average (SD)	Minimum	Maximum
WBC, 10^9^/L	9.56 (2.53)	5.64	20.28
NEU, 10^9^/L	3.55 (1.75)	1.32	11.46
LYM, 10^9^/L	5.24 (1.11)	3.17	7.73
MONO, 10^9^/L	0.57 (0.19)	0.19	1.18
EOS, 10^9^/L	0.11 (0.04)	0.05	0.26
BAS, 10^9^/L	0.08 (0.03)	0.03	0.20
RBC, 10^12^/L	9.21 (1.08)	6.61	11.74
HGB, g/dL	12.57 (1.19)	10.10	16.60
HCT %	37.31 (3.61)	29.40	48.30
MCV, fL	40.80 (3.74)	34.30	52.40
MCH, pg	13.73 (1.26)	11.60	17.70
MCHC, g/dL	33.69 (0.76)	31.90	34.80
RDW, %	23.18 (1.38)	20.60	27.60
PLT, 10^9^/L	641.77 (146.08)	350.00	1016.00
MPV, fL	4.80 (0.35)	4.10	5.70

^1^ WBC = white blood cell count, NEU = neutrophil count, LYM = lymphocyte count, MONO = monocyte count, EOS = eosinophil count, BAS = basophil count, RBC = red blood cell count, HGB = hemoglobin, HCT = hematocrit, MCV = mean corpuscular volume, MCH = mean corpuscular hemoglobin, MCHC = mean corpuscular hemoglobin concentration, RDW = red cell distribution width, PLT = platelets, and MPV = mean platelet volume.

**Table 3:** Associations between feed efficiency phenotypes and hematology parameters in beef heifers measured on d 42 of the study.

Trait	Hematology Parameter *	Reg ^†^	SE	*R* ^2^	*p* ^‡^
ADG	WBC	−0.0273	0.0141	0.2037	**0.0526**
	NEU	−0.0210	0.0212	0.1782	0.3208
	LYM	−0.0773	0.0305	0.2526	**0.0112**
	MONO	−0.1107	0.1885	0.1950	0.5571
	EOS	−0.5944	0.8276	0.1859	0.4727
	BAS	−1.9405	1.0354	0.2128	**0.0609**
	RBC	−0.0619	0.0361	0.2384	**0.0864**
	HGB	−0.0115	0.0289	0.1967	0.6914
	HCT	−0.0087	0.0096	0.2142	0.3642
	MCV	0.0113	0.0104	0.1947	0.2779
	MCH	0.0479	0.0291	0.2151	**0.0997**
	MCHC	0.1206	0.0439	0.3107	**0.0060**
	RDW	3.4880	2.6008	0.1944	0.1799
	PLT	−0.0001	0.0002	0.1741	0.5418
	MPV	0.1163	0.1090	0.2233	0.2860
ADFI	WBC	0.0081	0.0708	0.2217	0.9085
	NEU	0.0109	0.1027	0.2200	0.9156
	LYM	0.0100	0.1576	0.2135	0.9496
	MONO	0.2004	0.9160	0.2205	0.8268
	EOS	0.4040	4.0199	0.2194	0.9200
	BAS	−3.1918	5.0772	0.2204	0.5296
	RBC	−0.3916	0.1719	0.2885	**0.0227**
	HGB	−0.1979	0.1368	0.2527	0.1480
	HCT	−0.0847	0.0451	0.2760	**0.0602**
	MCV	0.0327	0.0512	0.2215	0.5227
	MCH	0.1614	0.1430	0.2328	0.2592
	MCHC	0.5074	0.2141	0.3062	**0.0178**
	RDW	9.9999	12.6317	0.2202	0.4286
	PLT	0.0006	0.0011	0.2303	0.6150
	MPV	0.1415	0.5340	0.2213	0.7910
G:F	WBC	−0.0035	0.0014	0.1849	**0.0134**
	NEU	−0.0030	0.0022	0.1214	0.1642
	LYM	−0.0010	0.0030	0.2511	**0.0014**
	MONO	−0.0152	0.0191	0.1349	0.4275
	EOS	−0.0978	0.0837	0.1096	0.2426
	BAS	−0.2222	0.1057	0.1570	**0.0355**
	RBC	−0.0033	0.0037	0.1293	0.3835
	HGB	0.0016	0.0030	0.1096	0.5976
	HCT	0.0002	0.0010	0.1084	0.8606
	MCV	0.0014	0.0010	0.1236	0.1932
	MCH	0.0049	0.0030	0.1392	**0.0958**
	MCHC	0.0086	0.0048	0.1755	**0.0715**
	RDW	0.2520	0.2712	0.1194	0.3527
	PLT	−2.8 × 10^−5^	2.3 × 10^−5^	0.1122	0.2173
	MPV	0.0150	0.0111	0.1880	0.1764

* WBC = white blood cell count, NEU = neutrophil count, LYM = lymphocyte count, MONO = monocyte count, EOS = eosinophil count, BAS = basophil count, RBC = red blood cell count, HGB = hemoglobin, HCT = hematocrit, MCV = mean corpuscular volume, MCH = mean corpuscular hemoglobin, MCHC = mean corpuscular hemoglobin concentration, RDW = red cell distribution width, PLT = platelets, and MPV = mean platelet volume. ^†^ Reg is the estimated increase in the trait per unit change in the hematology parameter. ^‡^ Nominal *p*-values are presented. *p*-values in bold represent those with *p* < 0.1.

**Table 4:** Differentially expressed genes associated with ADG and G:F in beef heifers. No DEGs were associated with ADFI.

Phenotype	Gene	Reg ^†^	Nominal *p*	FDR-Adjusted *p*
ADG	FAH	−0.5625	3.54 × 10^−6^	0.0543
	LOC104972586	−0.5400	1.02 × 10^−5^	0.0778
	COL1A2	0.5281	1.72 × 10^−5^	0.0879
G:F	COL1A2	0.5763	1.78 × 10^−6^	0.0273
	B9D1	0.5456	7.85 × 10^−6^	0.0601
	CCDC151	0.5267	1.82 × 10^−5^	0.0611
	SMARCA2	−0.5167	2.79 × 10^−5^	0.0611
	NEK2	0.5214	2.29 × 10^−5^	0.0611
	CIAPIN1	0.5221	2.22 × 10^−5^	0.0611
	RGS10	−0.5179	2.66 × 10^−5^	0.0611
	U2AF1	0.5084	3.94 × 10^−5^	0.0674
	XKR5	0.5083	3.96 × 10^−5^	0.0674
	NRP2	−0.4924	7.46 × 10^−5^	0.0923
	ATP6V0E2	0.4846	0.0001	0.0923
	PLOD1	−0.4869	9.20 × 10^−5^	0.0923
	UQCRFS1	0.4841	0.000102	0.0923
	LOC112443184	0.4904	8.06 × 10^−5^	0.0923
	BCL2A1	0.4902	8.11 × 10^−5^	0.0923
	FAH	−0.4897	8.27 × 10^−5^	0.0923
	LOC101904536	−0.4889	8.53 × 10^−5^	0.0923

^†^ Reg is the estimated increase in the trait per unit change in gene expression.

## Data Availability

The data presented in this study are openly available in the NCBI Sequence Read Archive (SRA) database, reference number PRJNA1153552. [NCBI Sequence Read Archive] [https://www.ncbi.nlm.nih.gov/bioproject/PRJNA1153552 (accessed on 6 May 2025)] [PRJNA1153552].

## Supplementary Materials

The supporting information can be downloaded at: https://www.mdpi.com/article/10.3390/ijms26104633/s1.
